# A Comparison of the Epidemiological Characteristics Between Influenza and COVID-19 Patients: A Retrospective, Observational Cohort Study

**DOI:** 10.7759/cureus.49280

**Published:** 2023-11-23

**Authors:** Omar Naji, Iman Darwish, Khaoula Bessame, Tejal Vaghela, Anja Hawkins, Mohamed Elsakka, Hema Merai, Jeremy Lowe, Miriam Schechter, Samuel Moses, Amanda Busby, Keith Sullivan, David Wellsted, Muhammad A Zamir, Hala Kandil

**Affiliations:** 1 Orthopaedics, West Hertfordshire Hospitals NHS Trust, Watford, GBR; 2 Internal Medicine, West Hertfordshire Hospitals NHS Trust, Watford, GBR; 3 Radiology, West Hertfordshire Hospitals NHS Trust, Watford, GBR; 4 Corporate Department, West Hertfordshire Hospitals NHS Trust, Watford, GBR; 5 Microbiology, West Hertfordshire Hospitals NHS Trust, Watford, GBR; 6 Virology, East Kent Hospitals University NHS Foundation, Kennington, GBR; 7 Health Research Methods Unit, University of Hertfordshire, Hatfield, GBR

**Keywords:** sars-cov-2 (severe acute respiratory syndrome coronavirus -2), chest radiograph, clinical epidemiology, seasonal influenza, covid-19

## Abstract

Background and objective

It is crucial to make early differentiation between coronavirus disease 2019 (COVID-19) and seasonal influenza infections at the time of a patient's presentation to the emergency department (ED). In light of this, this study aimed to identify key epidemiological, initial laboratory, and radiological differences that would enable early recognition during co-circulation.

Methods

This was a retrospective, observational cohort study. All adult patients presenting to our ED at the Watford General Hospital, UK, with a laboratory-confirmed diagnosis of COVID-19 (2019/20) or influenza (2018/19) infection were included in this study. Demographic, laboratory, and radiological data were collected. Binary logistic regression was employed to determine features associated with COVID-19 infection rather than influenza.

Results

Chest radiographs suggestive of viral pneumonitis and older age (≥80 years) were associated with increased odds of having COVID-19 [odds ratio (OR): 47.00, 95% confidence interval (CI): 21.63-102.13 and OR: 64.85, 95% CI: 19.96-210.69 respectively]. Low eosinophils (<0.02 x 10^9^/L) were found to increase the odds of COVID-19 (OR: 2.12, 95% CI: 1.44-3.10, p<0.001).

Conclusions

Gaining awareness about the epidemiological, biological, and radiologic presentation of influenza-like illness can be useful for clinicians in ED to differentiate between COVID-19 and influenza. This study showed that older age, eosinopenia, and radiographic evidence of viral pneumonitis significantly increase the odds of having COVID-19 compared to influenza. Further research is needed to determine if these findings are affected by acquired or natural immunity.

## Introduction

Both seasonal influenza and coronavirus disease 2019 (COVID-19) can cause life-threatening illness and death, especially in vulnerable populations [1,2]. It is postulated that non-pharmacological interventions such as lockdowns, social distancing, and face-coverings implemented to control the COVID-19 pandemic have led to an overall change in human behaviour [3] and resulted in a low level of influenza worldwide [4]. However, it seems probable that influenza will once again become a significant public health concern now that these pandemic management measures have been abandoned in the UK [5].

COVID-19 and influenza share similarities in their clinical presentation [6]. However, there are differences in the epidemiology of these two viruses, which influence both clinical and infection control management. Countries in the northern hemisphere closely monitor flu trends in Australia, because this helps predict the winter flu season in their own countries. In Australia, there has been a sharp increase in influenza notifications, which began earlier than usual this year and had a higher severity than the five-year average [5]. According to the latest weekly UK influenza and COVID-19 report, emergency department (ED) visits for acute respiratory infection have increased marginally and continued to be above average levels [7]. This data raises concerns about the potential for a COVID-19 and influenza twindemic, which can put further strain on already-stretched laboratory diagnostic capabilities [8]. Co-circulation of both viruses has been reported in 2022/23, which has caused a significant disease burden [9].

Early identification of these infections at the time of presentation to the hospital would enable the implementation of transmission-reducing measures and appropriate treatment regimens. As with COVID-19, influenza testing is necessary for clinical diagnosis and treatment. It is also important for infection prevention and control, and appropriate patient placement to prevent healthcare-associated infections and potential co-infection with other viruses, which could have serious effects. Conventional RT-PCR testing has a long turnaround time (usually >12 hours). Hence, this method cannot be relied upon to guide quick decision-making in the ED. Although point-of-care viral tests have the potential to speed up the diagnosis, their accessibility during winter can be limited due to high demand. Understanding key epidemiological, laboratory, and radiological characteristics of influenza and COVID-19 might aid in rapid ED decision-making [10].

Several studies have compared the epidemiology, clinical presentation, and outcomes of influenza and COVID-19 [11,12]. However, comparative data on the combined epidemiological, laboratory, and chest X-ray features at the time of admission is scarce. The aim of this study was to investigate if there are any significant differences between influenza and COVID-19 patients in their epidemiological characteristics, initial blood tests, and chest radiographs performed on admission to hospitals, which could help inform responses during periods of co-circulation.

## Materials and methods

Patient groups

This study involved two cohorts of patients. The first one included all adult patients (≥18 years) with laboratory-confirmed COVID-19 presenting to ED at the Watford General Hospital in March/April 2020. The second comprised all adults with laboratory-confirmed influenza virus presenting in October 2018-April 2019, and this timeframe was chosen to reduce potential bias caused by self-isolation and shielding policies implemented with the advent of COVID-19. Analysis was confined to hospital-admitted patients due to blood test availability. 

Demographics and comorbidities were collected from the Trust’s electronic patient information system (Sunquest® v8.3). The Pathology Manager was used to collect biochemical laboratory and microbiology data. This retrospective observational cohort study was reviewed by the West Hertfordshire Teaching Hospitals NHS Trust R & D Steering Group, which determined that no ethical approval was needed for this review involving routine clinical data, as per HRA guidance.

Laboratory and radiological data

Nose and throat swabs were collected from COVID-19-suspected patients. SARS-CoV-2 diagnoses were confirmed by RT-PCR. Viral co-infection was diagnosed by testing a second swab on BioFire® RP2. Influenza infection was diagnosed by testing nose and throat swabs on BioFire® RP2. Normal reference values for routine blood tests were as follows: white blood cell count: 3.2-10.5 x 10^9^/L; neutrophil count: 1.5-7.2 x 10^9^/L; lymphocyte count: 1.1-3.2 × 10^9^/L; and C-reactive protein (CRP): <5 mg/L. 

The Computerised Radiology Information System (CRIS) and Picture Archiving and Communication System (PACS) were used to filter chest radiographs alongside reports. Radiographs were interpreted by a team of radiologists blinded to the initial report. A third opinion was sought in cases of discrepancy between interpretations. The presenting chest radiograph was interpreted without reference to subsequent chest imaging to limit bias and to simulate the scenario experienced by the initial reporting radiologist. Comparison with previous chest radiographs, if available, was allowed, to help establish the radiographic baseline for each patient. Chest radiographs were initially categorized into two groups: radiographically normal and abnormal. Any abnormality found was further divided into the following four categories: (a) infective (likely viral), (b) infective (likely non-viral), (c) non-infective, and (d) dual pathology (infective and non-infective) causes. Radiographic findings of infective (likely viral) causes were either that of ground-glass opacification (haziness/increased lung opacity where vessels/airways within the lung parenchyma are still visualised) or dense consolidation bilaterally (homogenous opacification of the alveoli where vessels are hardly visualised). Ground-glass opacification is less opaque compared to consolidation [13]. Radiographic findings of infective (likely non-viral) mainly involved appearances suggestive of either typical bacterial (e.g., lobar consolidation in Streptococcus pneumoniae) or atypical infection (e.g., nodular consolidation in Mycoplasma pneumoniae or tuberculosis) [14]. Radiographic appearances of non-infective causes included various presentations, such as linear lung collapse, pulmonary congestion, and interstitial lung diseases [15].

Statistical analysis 

The primary outcome was COVID-19 compared to an influenza diagnosis. Univariable binary logistic regression was performed and evaluated using likelihood ratio tests. For categorical variables, groups were collated if appropriate. Blood test results were considered in continuous form and categorised into low/normal/high values, according to standard practice. Comorbidities were grouped by the presence of disease type (e.g., lung disease: asthma, COPD, bronchiectasis, restrictive lung disease, interstitial lung disease; cardiac diseases: ischaemic heart disease, congestive heart failure, atrial fibrillation). Variables were selected for multivariable modelling if p<0.2. Forward conditional multivariable binary logistic regression analyses were performed, retaining variables with p<0.05 (likelihood ratio tests). Interactions were investigated if clinically relevant.

Sensitivity analyses were performed for binary variables with >20% missing data by assuming that all missing data took one value and then the other. Akaike Information Criterion (AIC) values were compared to determine the most appropriate form for continuous variables and the proportion of variance explained by variables in the model by pseudo R-squared values. Analyses were conducted in Stata IC/15.1.

## Results

Demographics

A total of 826 and 273 laboratory-confirmed COVID-19 and influenza cases respectively presented to ED during the study period. A substantial increase in odds ratio (OR) was seen across age groups; those aged 40-59 years had an increased odds of a COVID-19 diagnosis [OR: 5.3, 95% confidence interval (CI): 2.9-9.5] compared to those aged 18-39 years. These odds also increased for the 60-79 age group (OR: 9.9, 95% CI: 5.5-17.8) and further still for the 80+ years age group (OR: 18.8, 95% CI: 9.8-36.0). Males were more likely than females to present with COVID-19 (OR: 1.42, 95% CI: 1.03-1.95, p=0.034). No difference in the odds of having influenza compared to COVID-19 was apparent between ethnic groups (p=0.306). However, there was a high level of missing ethnicity-related data across both groups: 8.5% for influenza and 17.4% for COVID-19. As there were few black or mixed/other ethnicities, the variable was recorded as white versus minority ethnic for modelling (Table 1).

**Table 1:** Univariable comparison of demographics and comorbidities between influenza and COVID-19 patients CI: confidence interval; COVID-19: coronavirus disease 2019

Characteristics		Influenza, n (%)	COVID-19, n (%)	Odds Ratio (95% CI)	P-value
		N=200	N=579		
Demographics					
Age Group (Years)	18-39	52 (26.0%)	21 (3.6%)	-	<0.001
	40-59	68 (34.0%)	145 (25.0%)	5.28 (2.95, 9.46)	
	60-79	54 (27.0%)	216 (37.3%)	9.9 (5.5, 17.83)	
	80+	26 (13.0%)	197 (34.0%)	18.76 (9.78, 35.98)	
Sex	Female	104 (52.0%)	251 (43.4%)	-	0.034
	Male	96 (48.0%)	328 (56.6%)	1.42 (1.03, 1.95)	
Ethnicity	White	135 (67.5%)	382 (66.0%)	-	0.306
	Asian	34 (17.0%)	64 (11.1%)	0.67 (0.42, 1.05)	
	Black	5 (2.5%)	15 (2.6%)	1.06 (0.38, 2.97)	
	Mixed/Other	9 (4.5%)	17 (2.9%)	0.67 (0.29, 1.53)	
	Unknown	17 (8.5%)	101 (17.4%)		
Comorbidities					
Lung Disease	None	114 (57.9%)	400 (69.1%)	-	0.004
	Present	83 (42.1%)	179 (30.9%)	0.61 (0.44, 0.86)	
Cardiac Disease	None	148 (75.1%)	403 (69.6%)	-	0.136
	Present	49 (24.9%)	176 (30.4%)	1.32 (0.91, 1.91)	
Neuro Disease	None	159 (80.7%)	476 (82.2%)	-	0.639
	Present	38 (19.3%)	103 (17.8%)	0.91 (0.6, 1.37)	
Diabetes	None	155 (78.7%)	436 (75.3%)	-	0.332
	Present	42 (21.3%)	143 (24.7%)	1.21 (0.82, 1.79)	
Hypertension	None	140 (71.1%)	288 (49.7%)	-	<0.001
	Present	57 (28.9%)	291 (50.3%)	2.48 (1.75, 3.52)	
Chronic Kidney Disease	None	184 (93.4%)	462 (79.8%)	-	<0.001
	Present	13 (6.6%)	117 (20.2%)	3.58 (1.97, 6.52)	
Cancer	None	175 (88.8%)	503 (86.9%)	-	0.470
	Present	22 (11.2%)	76 (13.1%)	1.20 (0.73, 1.99)	
Autoimmune Disease	None	180 (91.4%)	501 (86.5%)	-	0.064
	Present	17 (8.6%)	78 (13.5%)	1.65 (0.95, 2.86)	
Mental Health	None	155 (79.1%)	439 (78.4%)	-	0.839
	Present	41 (20.9%)	121 (21.6%)	1.04 (0.70, 1.55)	
Dementia	None	191 (97.4%)	466 (83.2%)	-	<0.001
	Present	5 (2.6%)	94 (16.8%)	7.71 (3.09, 19.24)	
Any Comorbidities	None	40 (20.3%)	102 (17.6%)	-	0.403
	Present	157 (79.7%)	477 (82.4%)	1.19 (0.79, 1.79)	
Comorbidity Count	0	40 (20.3%)	102 (17.6%)	1.20 (1.08, 1.32)	<0.001
	1-2	105 (53.3%)	219 (37.8%)		
	3-4	36 (18.3%)	172 (29.7%)		
	5+	16 (8.1%)	86 (14.9%)		
Smoking Status	Never	163 (82.7%)	473 (81.7%)	-	0.740
	Ever	34 (17.3%)	106 (18.3%)	1.07 (0.70, 1.64)	
Frailty Score	0	0 (0.0%)	94 (26.4%)	1.12 (1.05, 1.21)	0.001
	1-3	125 (63.8%)	60 (16.9%)		
	4-6	64 (32.7%)	143 (40.2%)		
	7-9	7 (3.6%)	59 (16.6%)		
	Missing	4	223	-	

Comorbidities

Hypertension, cardiac disease, chronic kidney disease (CKD), autoimmune disease, and dementia were each found to increase the odds of COVID-19 (Table 1, p<0.2). Conversely, lung disease was more prevalent among those with influenza (n=83, 42.1%) than COVID-19 (n=179, 30.9%) and hence it reduced the odds of a COVID-19 diagnosis (OR: 0.61, 95% CI: 0.44-0.86, p=0.004). For individual patients, a higher count of comorbidities increased the odds of COVID-19 compared to influenza but no clear pattern in the distribution could be seen. Notably, 40 (20.3%) influenza patients reported no comorbidities compared to 102 (17.6%) in the COVID-19 group. Although there seemed to be an association between increasing frailty score and the odds of having COVID-19, frailty scores were not included at the modelling stage since it was systematically missing in COVID-19 patients.

Radiographic findings

In the COVID-19-positive group, only 85 out of the 487 (17.5%) chest radiographs were reported as normal. Radiographic features of viral infection were reported in 333 (68.4%) chest radiographs, whereas 26 (5.6%) chest radiographs showed non-viral infective pathology and 38 (7.8%) demonstrated non-infective pathology. In addition, five (1.0%) illustrated dual pathology. 

Univariable analysis showed that chest radiographs suggestive of viral infection were associated with increased odds of a COVID-19 diagnosis compared to a clear chest radiograph (OR: 32.4, 95% CI: 16.6-63.3, p<0.001) (Table 2).

**Table 2:** Univariable comparison of radiological and laboratory findings between influenza and COVID-19 patients *P-values from likelihood ratio tests CI: confidence interval; COVID-19: coronavirus disease 2019; CRP: C-reactive protein; IQR: interquartile range

Findings		Influenza	COVID-19	Odds Ratio (95% CI)	P-value*
		N=200	N=579		
Radiological					
Chest X-ray Results, n (%)	Clear	91 (69.5%)	85 (17.5%)	-	<0.001
	Viral	11 (8.4%)	333 (68.4%)	32.41 (16.59, 63.30)	
	Non-viral	11 (8.4%)	26 (5.3%)	2.53 (1.18, 5.43)	
	Non-infective	18 (13.7%)	38 (7.8%)	2.26 (1.20, 4.26)	
	Both	0 (0.0%)	5 (1.0%)	-	
	Missing	69	92	-	
Blood test					
White Cell Count (10^9^/L)	Median (IQR)	7.3 (5.3, 10.2)	7.1 (5.4, 10.0)	0.99 (0.96, 1.03)	0.727
	N	173	519		
Platelet Count (10^9^/L)	Median (IQR)	194 (154, 241)	203 (154, 274)	1.00 (1.00, 1.00)	0.024
	N	173	518		
Neutrophils (Categorical), n (%)	Normal	114 (32.9%)	349 (33.6%)	-	0.744
	Neutrophilia >7.2 x 10^9^/L	59 (17.1%)	170 (16.4%)	0.94 (0.65, 1.35)	
Neutrophils (10^9^/L) (Continuous)	Median (IQR)	5.86 (3.66, 8.08)	5.55 (3.90, 8.24)	1.01 (0.96, 1.05)	0.800
	N	173	519		
Lymphocytes (Categorical), n (%)	Normal	92 (26.6%)	257 (24.8%)	-	0.404
	Lymphopenia <0.8 x 10^9^/L	81 (23.4%)	262 (25.2%)	1.16 (0.82, 1.63)	
Lymphocytes (10^9^/L) (Continuous)	Median (IQR)	0.83 (0.54, 1.24)	0.80 (0.54, 1.13)	0.99 (0.92, 1.06)	0.756
	N	173	519		
Eosinophils (Categorical), n (%)	Eosinopenia <0.02 x 10^9^/L	112 (65.5%)	414 (80.1%)	2.12 (1.44, 3.10)	<0.001
	Normal	59 (34.5%)	103 (19.9%)	-	
	Eosinophilia >0.5 × 10^9^/L	2 (1.0%)	2 (0.3%)	0.27 (0.04, 1.94)	
Eosinophils (10^9^/L) (continuous)	Median (IQR)	0.01 (0.00, 0.04)	0.00 (0.00, 0.02)	0.12 (0.02, 1.02)	0.046
	N	173	519		
CRP (Categorical), n (%)	<100 mg/L	163 (80.7%)	355 (61.1%)	2.78 (1.87, 4.12)	<0.001
	≥100 mg/L	37 (18.3%)	224 (38.6%)		
CRP (mg/L) (Continuous)	Median (IQR)	43.1 (19.0, 100.0)	90.0 (49.1, 153.0)	1.00 (1.00, 1.01)	<0.001
	N	147	500		
Creatinine (mg/dL)	Median (IQR)	69.0 (53.5, 79.5)	59.0 (41.0, 73.0)	0.98 (0.97, 0.99)	<0.001
	N	124	431		

Laboratory investigations

Low eosinophil count as a continuous measure increased the odds of COVID-19 but a marked difference was seen when categorising patients into those with eosinopenia (<0.02 x 10^9^/L) compared to normal levels (OR: 2.12, 95% CI: 1.44-3.10, p<0.001). All patients had elevated CRP levels (above 4 mg/L) but those above 100 mg/L had increased odds of COVID-19 (OR: 2.78, 95% CI: 1.87-4.12, p<0.001). There was a difference in platelet count between cohorts but with no observed effect on the odds of having COVID-19 compared to influenza (OR: 1.00, 95% CI: 1.00-1.00, p=0.024) (Table 2).

Modelling results

The variables included in modelling (if p<0.2) were age, sex, chest radiographs, C-reactive protein (continuous and categorical), creatinine, platelet count, eosinophils (continuous and categorical), dementia, diabetes, CKD, hypertension, lung disease, cardiac disease, autoimmune disease and comorbidity count.

The odds of COVID-19 increased with a chest radiograph showing evidence of viral pneumonitis (OR: 47.0, 95% CI: 21.6-102.1, p<0.001) compared to a clear chest radiograph, an increase in odds compared to the univariable analysis once adjusted for other variables. Other types of abnormal chest radiographs (non-viral, non-infective or both) had a combined effect of increasing odds to a lesser effect (OR: 2.06, 95% CI 1.10-3.89, p=0.025) (Table 2).

Increasing age groups increased the odds of COVID-19 compared to influenza. A similar effect was seen in the univariable analysis for age groups of 40-59 (OR: 4.83, 95% CI: 1.91-12.23, p=0.001) and 60-79 years (OR: 10.78, 95% CI: 4.07-28.61, p<0.001) compared to those in the 18-39 age group. However, adjusting for other covariates in the model increased the odds ratio for the 80+ years group (OR: 64.85, 95% CI: 19.96-210.69, p<0.001).

Cardiac disease was more prevalent in patients with hypertension: 174 out of 348 (50%) hypertensive patients had cardiac disease compared to 51 out of 428 (12%) patients without hypertension. Therefore, the association between cardiac disease and hypertension was assessed, with evidence for its inclusion (p=0.0008). Cardiac disease without hypertension was associated with decreased odds of influenza (OR: 0.10, 95% CI: 0.03-0.30). However, for patients with hypertension, no association with cardiac disease was found. Lung disease was more common in influenza patients, irrespective of whether they were hypertensive or not (OR: 0.37, 95% CI: 0.21-0.64, p=0.001) (Table 3).

**Table 3:** Results for the binary logistic regression model (n=618, pseudo R2=0.4432) CI: confidence interval

Characteristics		Odds Ratio (95% CI)	P-value
Chest X-ray	Clear	-	-
	Viral	47.00 (21.63, 102.13)	<0.001
	Other	2.06 (1.10, 3.89)	0.025
Age Group (Years)	18-39	-	-
	40-59	4.83 (1.91, 12.23)	0.001
	60-79	10.78 (4.07, 28.61)	<0.001
	80+	64.85 (19.96, 210.69)	<0.001
Lung Disease		0.37 (0.21, 0.64)	0.001
Cardiac Disease		0.1 (0.03, 0.3)	<0.001
Hypertension (HTN)		0.89 (0.42, 1.87)	0.757
Cardiac/HTN interaction		9.49 (2.48, 36.36)	0.001

Sensitivity analyses

There were high levels of missing data for both chest radiograph results and ethnicity. Re-fitting the final model by firstly assuming all missing chest radiograph results were suggestive of viral infection, and then secondly assuming they were clear/normal reduced the OR for chest radiographs suggestive of viral infection, but the increased odds of COVID-19 were still strongly apparent in both cases. Assuming all missing ethnicity values were firstly white and then of minor ethnicity had no effect on its association with the odds of having COVID-19 and hence ethnicity values did not merit inclusion in the model.

## Discussion

Our results showed that certain sets of demographic, laboratory, and radiological indicators are associated with increased odds of COVID-19 versus influenza diagnosis. These indicators can guide clinicians to implement appropriate actions while awaiting confirmatory laboratory diagnosis of infection.

In line with the national pattern [16,17], our study showed that males are more affected by COVID-19 than females and that risk increases with age. The underlying mechanism of this gender difference is unclear but may be attributed to the differential regulation of innate and adaptive immune responses, which, in turn, regulates sex-biased pathogenesis and mortality with regard to various pathogens [18]. Similarly, the age trend in our cohort reflects the national influenza activity during the 2018/19 winter season. The health impact of influenza infection nationally was also predominantly seen in younger age groups with both hospitalisations and ICU admissions in the 2018/19 season seen particularly in the 15-44 and 45-64 age groups. The peak admission trends during 2018/19 were similar or higher than those observed in the previous seven seasons [19]. This suggests that data on influenza in this study is not particular to this season and indeed not much different from any other year since the introduction of influenza reporting schemes. The difference in age between cohorts is unlikely to relate to influenza vaccination trends among the elderly. Although winter 2018/19 saw the introduction of the new adjuvanted influenza vaccine for those aged ≥65 years, influenza vaccine uptake in England was slightly lower than the previous season across all ages [19].

Even though some ethnicity-related data were missing, COVID-19 patients in our cohort were predominantly of Caucasian backgrounds, which is in line with nationwide data during the pandemic. National cumulative data from the end of January to the beginning of May 2020 showed that 82% of COVID-19-infected patients were white [20]. A CDC study on ethnic disparities found that people from racial/minority ethnic groups are at a higher risk for hospitalisation with influenza [21], while in our cohort, the majority of patients admitted with influenza were Caucasians, reflecting the local population patterns.

Comorbid conditions appear to increase the risk for both COVID-19 and influenza infections. Older age and a range of underlying health conditions have been associated with an increased risk of severe influenza and associated mortality [22]. Several studies have previously provided information on individuals hospitalised with COVID-19 but the prevalence of comorbidities has varied, and this variation is seen across different geographical areas [23,24]. Interestingly, in a study [25] involving 34,128 patients, the characteristics of COVID-19 patients were described and compared to influenza-positive patients from previous years. Consistent differences were noted in the prevalence of respiratory disease, cardiovascular disease, and dementia, each more common among those hospitalised with influenza. Those hospitalised with COVID-19 were consistently seen to be less likely to have COPD, cardiovascular disease, and dementia than those hospitalised with influenza in recent years. Our findings also illustrate the variation in patient characteristics. 

In line with the International Severe Acute Respiratory and Emerging Infections Consortium (ISARIC) [16], our study found that the most prevalent comorbidities in COVID-19 patients were hypertension, cardiac disease, and lung disease. The differences in the prevalence of underlying respiratory disease between influenza and COVID-19 are particularly interesting, corroborating other studies [25]. Interestingly, the incidence of eosinopenia rather than lymphopenia was significantly different between cohorts. High CRP (>100 mg/L) was more prevalent in COVID-19 patients, although no differences remained once adjusted for other covariates. The combination of eosinopenia and high CRP was suggested by Li et al. as an indicator to differentiate between suspected COVID-19 patients and other patients attending clinics with COVID-19-like initial symptoms [26]. Andreozzi et al. showed that eosinopenia is higher on admission among COVID-19 than influenza patients [27]. 

The pathophysiology of eosinopenia in COVID-19 remains unclear, but it may be multifactorial. The mechanisms may include inhibition of eosinophil egress from the bone marrow, blockade of eosinophilopoiesis, reduced expression of chemokine receptors/adhesion factors, and/or direct eosinophil apoptosis induced by type 1 interferons released during the acute infection [28]. This study focused on the chest radiograph interpretation of COVID-19 and influenza cases [29] since chest radiographs are the most common imaging modality performed in ED, easily interpreted by clinicians. Our results suggest that COVID-19 patients are more likely to present with abnormal chest radiographs than influenza-positive patients. This is partly explained by the relatively late presentation of COVID-19 patients when abnormal radiographic appearances would be expected.

Our findings align with current literature; a study comparing clinical, biochemical, and radiographical findings in influenza and COVID-19 patients found a longer median time of symptom onset to radiological investigations (CT scan) in the COVID-19 group and more parenchymal lung involvement, compared to those of the influenza group [30]. Additionally, clinical aggravation of symptoms requiring hospitalisation often presents later in COVID-19 than in the influenza virus, enabling chest radiograph changes to become evident [31]. Our study did not collect data on the date of symptom onset to verify this. However, a systematic review comparing COVID-19 and influenza radiographs found that 84% of COVID-19 patients had abnormal chest radiographs compared to patients with influenza A (57%) or influenza B (33%) [32]. Goel et al. [33] found no differences in the chest radiograph features between COVID-19 and influenza patients; however, unlike in our cohort, it was unclear how the diagnosis of influenza was made, leaving the possibility that some patients in this cohort had varying viral diagnoses. 

Limitations

This study has several limitations. As this study was retrospective, some of the data related to comorbidity and ethnicity were lacking. Only patients with a laboratory-confirmed COVID-19 or influenza diagnosis were included in this study, thereby excluding positive patients lacking laboratory confirmation. Additionally, data were confined to patients from the first wave of the pandemic. Since then, several SARS-CoV-2 variants have emerged with multiple changes in the genetic sequence that can potentially change its pathogenicity and subsequently affect the individual response to the infection. However, age and the presence of comorbidities such as hypertension, obesity, cardiovascular disease, immunosuppression, smoking, and diabetes mellitus are more important predictors of severity, hospitalization, and mortality than SARS-CoV-2 variants. Although infection with Omicron is associated with a lower risk of severity compared to infection with the previous variants of concern [34], the epidemiological [35,36], clinical [37,38], laboratory [36,39,40], and radiological [41] presentation is overall similar across different severity groups. Another limitation is that we did not study the impact of vaccination on radiological and laboratory findings. Although protection from natural immunity or vaccination reduces the severity of re-infection, some people can experience more severe illness during re-infection, and across different severity groups, the clinical presentation is overall similar. However, it is unknown whether immune status impacts the laboratory and radiological presentation [37,38].

Our study assessed characteristics of individuals with COVID-19 and influenza on ED admission, a time when decisions regarding clinical and infection prevention management are urgently needed; however, this may not reflect the whole picture. Furthermore, we did not collect data on the date of symptom onset, which may explain some differences between cohorts, particularly relating to blood results and chest radiographs. However, this information is rarely accurate and frequently assumed or not provided. Furthermore, the generalisability of the results is limited by the fact that this was a single-centre study.

The strengths of the study include the inclusion of a large number of participants and the comprehensive comparison of influenza and COVID-19 patient characteristics. Another strength pertains to comparing the radiological findings on chest X-ray rather than CT. While CT is more sensitive and provides more details, chest X-ray is a very convenient and valuable tool used routinely in ED to evaluate chest diseases and inform management decisions [42].

## Conclusions

This study engaged in a comparative analysis of COVID-19 and influenza patient characteristics and investigations at a time when physicians in the ED are frequently making management decisions. Based on our findings, older age and radiographic evidence of viral pneumonitis significantly increase the odds of having COVID-19 compared to influenza infection. The present study may be considered a further step forward in the attempt to understand key differences between influenza and COVID-19 diseases. However, further studies are needed to provide insights into the impact of other variants of SARS-CoV-2 as well as COVID-19 vaccination on these characteristics.
